# Birth preparedness and complication readiness among pregnant women in Ethiopia: a systematic review and Meta-analysis

**DOI:** 10.1186/s12978-018-0624-2

**Published:** 2018-10-29

**Authors:** Abadi Kidanemariam Berhe, Achenef Asmamaw Muche, Gedefaw Abeje Fekadu, Getachew Mullu Kassa

**Affiliations:** 1 College of Medicine and Health Sciences, Adigrat University, Tigray, Ethiopia; 2 Department of Epidemiology and Biostatistics, Institute of Public Health, University of Gondar, Gondar, Ethiopia; 3 School of Public Health, College of Medicine and Health Sciences, Bahir Dar University, P.O.Box 79, Bahir Dar, Ethiopia; 4 College of Health Sciences, Debre Markos University, Debre Markos, Ethiopia

**Keywords:** Birth preparedness, Complication readiness, Ethiopia, Systemic review and Meta-analysis

## Abstract

**ᅟ:**

Birth preparedness and complication readiness is an essential component of safe motherhood programs that promote appropriate utilization of skilled maternal and neonatal care. Preparing for childbirth and its probable complications can reduce delays in seeking care. In Ethiopia, there were limited data on birth preparedness and complication readiness at the national level except a small scale studies conducted.This systemic review and meta-analysis study was conducted to assess the national estimates regarding the status of birth preparedness and complication readiness among pregnant women in Ethiopia.

**Methods:**

Preferred Reporting Items for Systematic Reviews and Meta-Analyses (PRISMA) guideline was followed during systemic review and meta-anaysis. The databases used to identify studies were; MEDLINE, PubMed, Google scholar, CINAHL, EMBASE and African Journals Online. Appropriate search terms were used to retrieve published studies conducted in Ethiopia. Joanna Briggs Institute Meta-Analysis of Statistics Assessment and Review Instrument (JBI-MAStARI) was used for critical appraisal of studies. The meta-analysis was conducted using STATA 14 software. Forest plots were used to present the findings of this meta-analysis. The *I*^2^ test statistics and Egger’s test were used to test heterogeneity and publication bias respectively. The pooled prevalence with 95% confidence intervals (CI) was computed. Duval and Tweedie nonparametric trim and fill analysis using the random-effect analysis was conducted to account for publication bias and high heterogeneity.

**Result:**

Thirteen studies and six thousand four hundred ninety three participants were included in this meta-analysis and overall pooled result showed 32% with 95% (25.6, 38.5) of the pregnant women were prepared for birth and its complications. In addition, 51.35% of women save money for birth and emergency case, 38.74% women identified skilled birth attendant, and only 26.33% of pregnant women were aware of danger signs during pregnancy. One fifth (20.59%) of women arranged transportation and 54.85% of women identified the place of birth. Only 8.18%pregnant women identified potential blood donor for emergency cases.

**Conclusion:**

Low proportions of pregnant women were prepared for childbirth and its complications. The Ministry of health, Regional health bureaus, health facilities and other stakeholders should work to improve birth preparedness and complication readiness among pregnant women in Ethiopia.

## Plain English summary

Delay in responding to the onset of labor and of its complications has a major barrier on reduction of maternal mortality and morbidity. Ethiopia is one of the countries with high maternal mortality, the country introduced different strategies to reduce maternal mortality like free maternal service, employed trained health extension worker at community level, strengthening midwifery professional training. Birth preparedness and complication readiness is nationally endorsed as an essential component of safe motherhood programs to reduce maternal and newborn mortality.

There were different studies conducted in different regions on birth preparedness and complication readiness but there is limited data on the pooled estimates at the national level. There fore the aim of this systematic review and meta analysis was to assess birth preparedness and complication readiness at nationally level in Ethiopia. Thriteen studies were included in this metaanalysis. Only one third of pregnant women were prepared for birth and ready for complication in ethiopia. Specifically this study showed 51.35% of women saving money, 38.74% women identified skilled birth attendant and 26.33% of pregnant women were aware of danger signs during pregnancy. One fifth of women arranged transportation and 54.85% of women identify the place of birth. Only 8.18% pregnant women identified potential blood donor for the case of emergency. Ministry of health, Regional health bureaus, health facilities and other stakeholders should work to improve this low birth preparedness and complication readiness among pregnant women in Ethiopia.

## Background

Globally, eight hundred women die every day due to pregnancy or child birth related complications. Almost all maternal deaths (99%) occur in developing countries, and more than half of this deaths occur in Sub-Saharan Africa [1]. Many of the complications that result in maternal mortality and perinatal deaths like abortion complications, ruptured uterus, puerperal sepsis, postpartum hemorrhage, and preeclampsia/eclampsia are unpredictable, and their onset can be both sudden and severe [2]. Delay in responding to the onset of labor and onset of complications by individual pregnant women, family and health care provider has been shown as major barrier to reducing maternal mortality and morbidity [3].

In many societies, cultural beliefs and lack of awareness inhibit preparation for delivery and seeking care. Due to this reason complications occur in unprepared family could take lots of time in understanding the problem; to get organize, in getting money, in finding transport and reaching the appropriate referral facility. Therefore, this delay in the decision, in reaching health facility and delay in receiving care can be solved by proper use of birth preparedness and complication readiness plan [4–6].

Ethiopia is one of the countries with high maternal mortality ratio (MMR), although the country is striving hard to reducing maternal mortality [7, 8]. In the last fifteen years; maternal mortality decreased from 871 to 412 per 100,000 live births due to the substantial effort made in improving maternal health services for pregnant and laboring women [9, 10]. Ethiopia did not achieve the Millennium Development Goal (MDG) which aimed to reduce the MMR to 267 maternal deaths per100,000 live births [11, 12]. Birth preparedness and complication readiness (BPCR) helps pregnant women how to identify skilled birth attendant, how to identify signs of labor and in recognizing danger signs for pregnancy-related complications. It also helps toreduce other barriers to seeking care, such as transport costs, perceptions of poor quality of care and cultural differences [13].

BPCR components are included in the new World Health Organization (WHO) model for antenatal care as part of antenatal care education in clinic setting [14]. Birth Preparedness and Complication Readiness is one of the strategy to promote timely utilization of skilled maternal and neonatal care timely. Preparing for childbirth and associated complications reduces the three delays of maternal death; delay in recognizine danger signs and decision of seeking care in home, delay in reaching health facility and delay in receiving health care by encouraging pregnant women, their families, and communities to effectively plan for births and prepare for emergencies if they occur [15, 16].

Even though BPCR is nationally endorsed as an essential component of safe motherhood programs in West Showa Zone (WSZ), Ethiopia in 1998 [17] low proportion of women are delivering at health facility (26%) [9] and high proportion of women are suffering from complications related to pregnancy and child birth (28.5%) [18–20]. Additionally there is limited data about BPCR plan of pregnant women’s at the national level, Therefore the aim of this systemic review and meta analysis is to assess mangitude of birth preparedness and complication readiness in Ethiopia and this will helps for policy makers and program coordinators, health professional and other stakeholders who are working on maternal health to have up-to-date information on the status of BPCR at national level.

## Methods

### Study design and search strategy

A systemic review and meta-analysis on birth preparedness and complication readiness in Ethiopia was done using published research articles. Major data bases like; MEDLINE, PubMed, Google scholar, CINAHL, EMBASE and African Journals Online were used to review published studies. All published articles up to August/2017 were included in the review. The reference lists of already identified studies were also searched to retrieve additional articles. The search was done using the following search terms; *“Birth preparedness”, “complication readiness”; “pregnant women”, “pregnant women identified skilled birth attendant”, “pregnant women saving money for emergency cases”, “pregnant women identifying potential blood donor”, “pregnant women arrangement for transportation”, “pregnant women identifying the place of delivery”, Ethiopia* separately and/or in combination using the Boolean operators like “OR” or “AND”.

### Study selection and eligibility criteria

Inclusion criteria:

❖ Participants: this review included studies conducted on birth preparedness and complication readiness among pregnant women in Ethiopia.

❖ Setting: Studies conducted both at community or institution level.

❖ Article which includes Birth preparedness and complication readiness as outcome

❖ Time frame: all studies irrespective of time of data collection or publication year

❖ Publication types: journal articles

❖ Studies published only in the English language were included in the review

Exclusion criteria:

❖ Studies which did not reported the outcome variable of this review.

❖ Repetitive publication

❖ Studies which did not specify study population and qualitative studies were excluded from systematic review and Meta-analysis

### Data extraction and data collection

Authors jointly prepare and the determined data extraction tool for this study. The tool includes questions on; the name of the author, publication year, study design, data collection period, sample size, study area, participants, response rate and prevalence of birth preparedness and complication readiness. Additionally,the tool contains information on; percentage of women who saved money for birth and emergency case, women who prepared blood donor, women who identified skilled birth attendant, women who were aware of danger signs during pregnancy, women who arranged transportation, women who identified place of birth and women who planed health facility delivery.All authors involved in the data extraction.

### Definitions of birth prepardness and complication readiness

Birth preparedness and complication readiness is measured by key elements. The key elements includes; arrangement for transportation, saving money for delivery, identify skilled birth attendant, identifying place of delivery and identifying blood donor for the case of emergency [3, 15, 21]. All studies that used the above definition of birth preparedness and complication readiness were included in this review.

### Quality assessment

Articles were reviewed using titles, abstracts and full review. Studies that did not meet inclusion criteria were excluded. Full texts of included studies were examined using the Joanna Briggs Institute Meta-Analysis of Statistics Assessment and Review Instrument (JBI-MAStARI) for critical appraisal.

### Publication bias and heterogeneity

Publication bias and heterogeneity were assessed using the Egger’s test and *I*^*2*^statistics, respectively [22]. Statistical significant publication bias was declared at *p*-value less than 0.05. The heterogeneity of studies was tested using *I*^*2*^ test statistics.*I*^*2*^ test statistics result of 25%, 50%, and 75% was declared as low, moderate and high heterogeneity respectively [23]. Random effect model was used for meta-analysis test result which indicated statistically significant heterogeneity [24]. For studies which showed the presence of publication bias, the Duval and Tweedie nonparametric trim and fill analysis was conducted to account for publication bias [25].

### Statistical methods and analysis

The analysis was conducted using STATA version 14. Forest plots were used to present the combined prevalence estimate with the 95% confidence interval (CI). For studies which didn’t present standard error (SE), it was calculated using the formula; SE = √p x (1-p)/n in Microsoft excel.The calculated SE and prevalence rate of each study was then entered into STATA software to calculate the overall prevalence and its 95% CI. The key elements of birth preparedness and complication readiness were presented using separate forest plots.

## Results

### Studies included in the meta-analysis

**Fig. 1:**
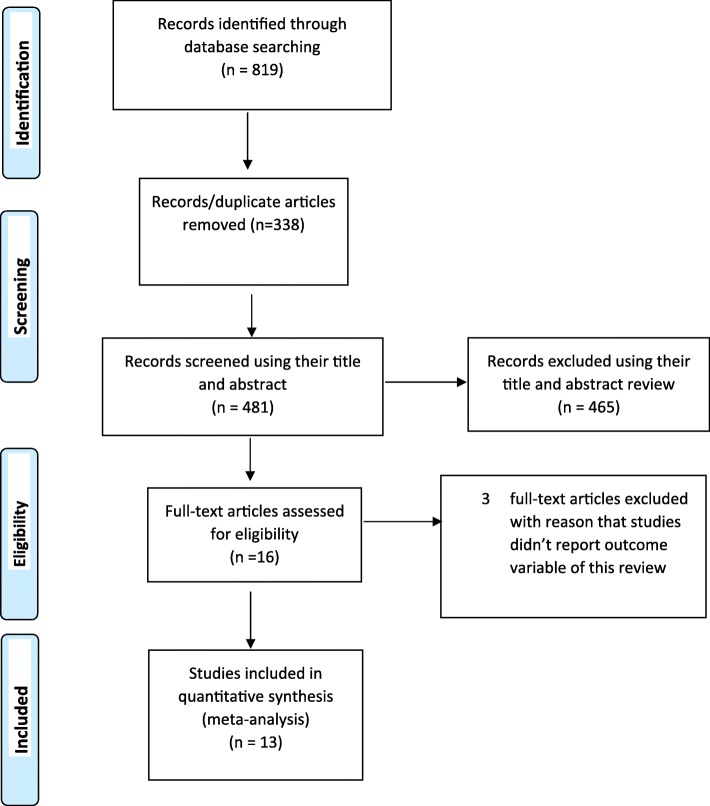
Flow diagram of the studies included in the Meta-analysis

A total of 819 research articles were identified by electronic search in MEDLINE, PubMed, Google scholar, CINAHL, EMBASE and African Journals Online data bases. Of which, 338were excluded due to duplication, 403through review of titles and 62 through reviewing of abstracts. Additionally 3full-text articles were excluded for not reporting the outcome variable and not including pregnant women as study population. Finally, 13 studies were found to be eligible and included in the Meta-analysis (Fig. 1).

The sample size of included in this review were totally 6493 which varied among studies included from 220 in Mekelle, Tigray regional state [26] to 819 in SouthWollo, Amhara regional state [27].Four of the included studies were from SNNP [28–31], 2 studies from Tigray Region [26, 32], 2 studies from Amhara Region [27, 33], 2 studies from Oromia Region [34, 35], 1 study from Dire Dawa city administration [36], 1 study from Harari Region [37] and 1 study from Addis Ababa city [38]. Additionally all studies included in the review were crossectional study designs (Table 1).

**Table 1:** Summary characteristics of studies included in study of birth preparedness and complication readiness among pregnant women in Ethiopia

S.No	Authors	Study year	Study design	Sample size	Study area	BPCR (95% CI)
1.	Begashaw et al.	2017	Cross-sectional	392	Mizan-Tepi University Teaching Hospital**,** SNNP Region	41.1 [36.23, 45.97]
2.		2016	Cross-sectional	405	Dire Dawa city	54.7 [49.85, 59.55]
3.	Bitew et al.	2016	Cross-sectional	819	SouthWollo, Amhara Region	24.1 [21.17, 27.03]
4.	Berhanu D. Isaac et al.	2016	Cross-sectional	423	Dere Teyara District, Harari Region	42.8 [38.08, 47.52]
5.	Dimtsu and Bugssa	2014	Cross-sectional	220	Mekelle, Tigray Region	32.3 [26.12, 38.48]
6.	Gebre et al.	2015	Cross-sectional	578	Wolayta Zone, SNNP Region	18.3 [15.15, 21.45]
7.	Hailu et al.	2011	Cross-sectional	743	Sidama Zone, SNNP Region	17 [14.30, 19.70]
8.	Hiluf and Fantahun	2008	Cross-sectional	534	Adigrat, Tigray Region	22 [18.48, 25.51]
9.	Kaso and Addisse	2014	Cross-sectional	575	Robe district, Oromia Region	16.5 [13.46, 19.53]
10.	Markos and Bogale	2014	Cross-sectional	580	Goba district, Oromia Region	29.9 [26.17, 33.63]
11.	Tiku	2015	Cross-sectional	224	Addiss Ababa City	56.3 [49.80, 62.79]
12.	Wuhib Bishaw et al.	2014	Cross-sectional	546	Basoliben District, Amhara Region	26.9 [23.18, 30.62]
13.	Zepre and Kaba	2017	Cross-sectional	454	Abeshige district, SNNP Region	37.20 [32.75, 41.65]

### Birth preparedness and complication readiness among pregnant women in Ethiopia

**Fig. 2:**
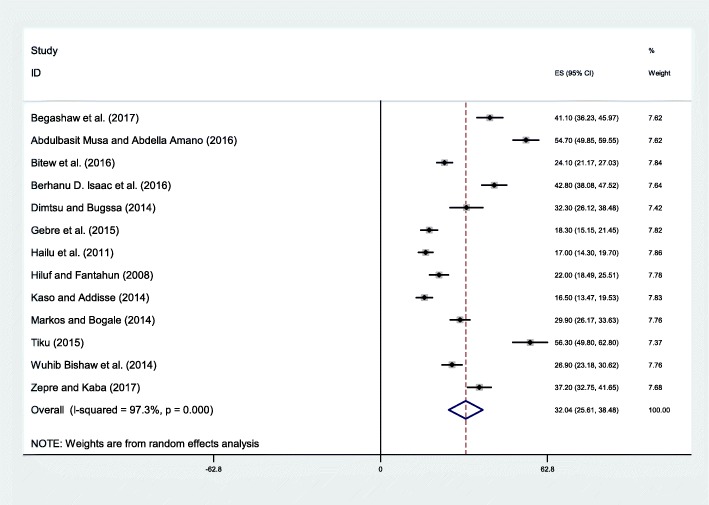
Forest plot displaying the pooled result of birth preparedness and complication readiness in Ethiopia

The minimum birth preparedness and complication readiness was 16.5% seen in a study conducted Robe district, Oromia Region [34] whereas the maximum was 56.3% observed in federal police referral hospital, Addis Ababa city [38]. The pooled result of the studies showed that only one third (32.04%; 95% CI: 25.61, 38.48) of pregnant women were prepared for birth and its complication (Fig. 2).The *I*^*2*^test statistics result showed high heterogeneity (I^2=^97.3%, p = < 0.001) and Eggers test (*p*-value < 0.001). Duval and Tweedie nonparametric trim and fill analysis using the random-effect analysis was conducted to account for publication bias and heterogeneity.

**Fig. 3:**
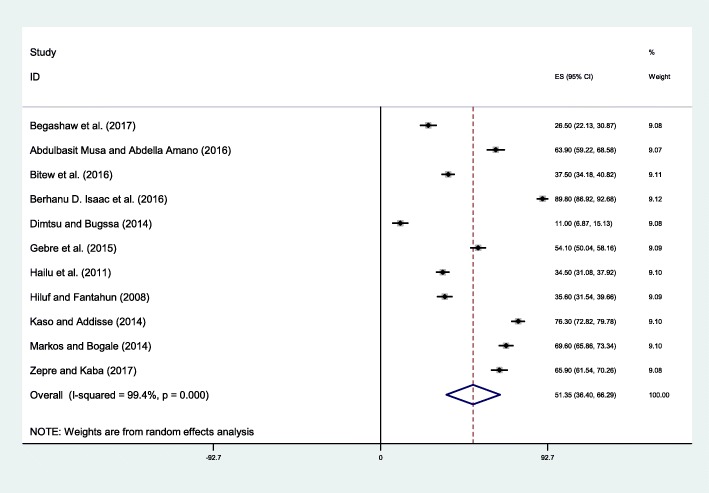
Forest plot displaying the pooled result of pregnant women save money for birth preparedness and complication readiness in Ethiopia

The lowest percentage of women in saving money was 11% observed in Mekelle, Tigray region [26] and the highest was 89.8% in Dere Teyara District, Harari [37]. The pooled meta-analysis showed that 51.35% (95% CI: 36.40, 66.29) of pregnant women saved money for childbirth and emergency case (Fig. 3). The *I*^*2*^test result showed high heterogeneity (I^2=^99.4%, *p* = < 0.001) but Egger’s test showed no statistical significant publication bias (*p*-value = 0.171).

**Fig. 4:**
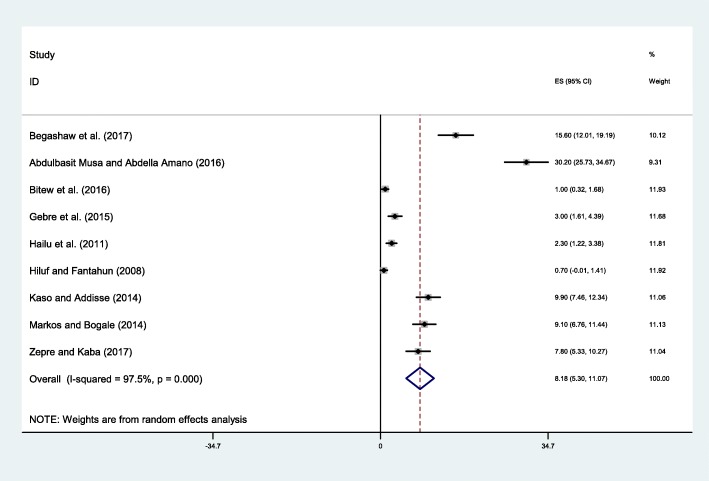
Forest plot displaying the pooled result of pregnant women prepared blood donor for emergency case during pregnancy and child birth in Ethiopia

In this sub analysis the heterogeneity test showed the presence of heterogeneity (*I*^*2*^ = 97.5%, *p* ≤ 0.001) and publication bias (Egger’s test *p*-value < 0.001). Therefore, the Duval and Tweedie nonparametric trim and fill analysis using the random-effect analysis was conducted to account for publication bias. Accordingly, the pooled result of studies showed only 8.18% (95% CI: 5.30, 11.07) of women have prepared potential blood donor for emergency case that may occurred during pregnancy and childbirth (Fig. 4).

**Fig. 5:**
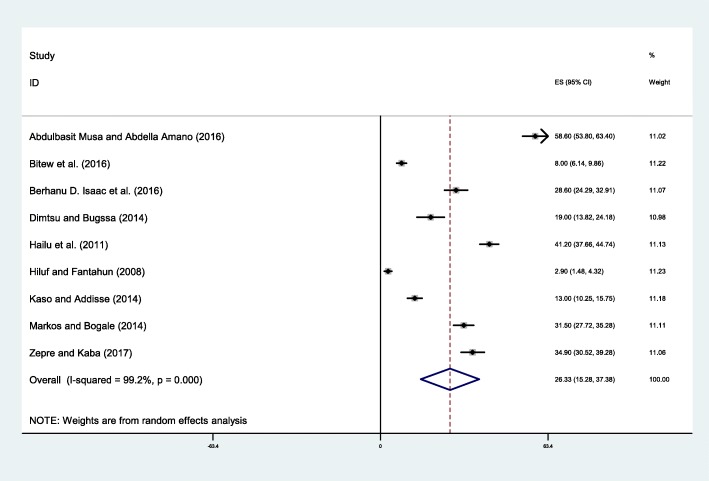
Forest plot displaying the pooled result of pregnant women aware of danger sign during pregnancy in Ethiopia

The lower awarness of danger sign of pregnancy was 2.80% obseved in a study conducted adigrat, Tigray region [32] where as the maximum awareness was 58.60% in Dilchora Referral Hospital, Dire Dawa city [36]. Finding of this study showed that only 26.33% (95% CI: 15.28, 37.38) of pregnant women were aware of danger signs of pregnancy (Fig. 5). The *I*^*2*^test result showed high heterogeneity (I^2=^99.2%, *p* < 0.001) and Eggers test (*p*-value < 0.01).

**Fig. 6:**
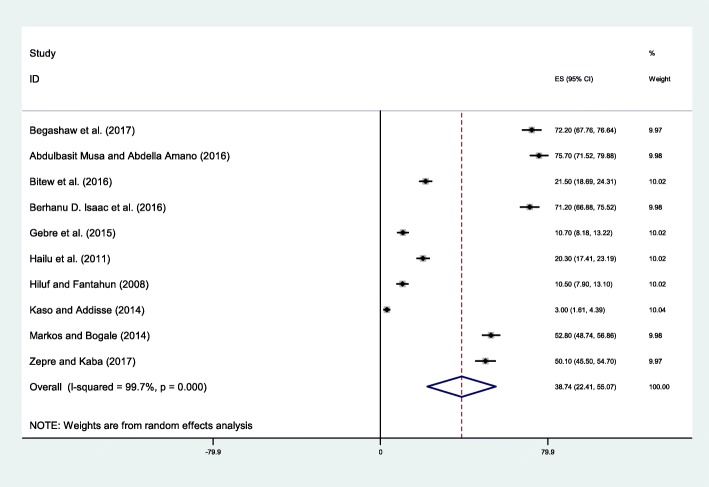
Forest plot displaying the pooled result of pregnant women identify skilled birth attendant for delivery in Ethiopia

The lowest percentage of pregnant women who identified skilled birth attendant was 3.0% which was a study conducted in Robe district, Oromia region [34].Findings of this study showed that only 38.74% (95% CI: 22.41, 55.07) pregnant women identified skilled birth attendant for delivery (Fig. 6). The *I*^*2*^test result showed high heterogeneity (I^2=^99.7%, *p* < 0.001) and Eggers test (*p*-value < 0.001).

There was difference in arrangement of transport as BPCR across regions; the lower magnitude 8.53% observed in Tigray region and maximum 88.70% in Dire Dawa city. The overall pooled result of this study showed only that one third (33.93%) of the pregnant women were arranged transport for birth and emergency case during pregnancy; there was wide variation across the studies conducted in different regions (Table 2). The*I*^*2*^test result showed high heterogeneity (I^2=^99.7%, *p* < 0.001) and Eggers test (*p*-value < 0.001).

**Table 2:** Percent of pregnant women who arranged transport as birth preparedness and complication readiness plan in Ethiopia

Study area	Number of studies	Estimation (95% CI)
SNNP Region	4	33.59 (10.56, 56.63)
Dire Dawa city	1	88.70 (85.62, 91.78)
Harari Region	1	30.50 (26.11, 34.89)
Oromia Region	2	45.64 (12.03, 79.26)
Amhara Region	1	10.80 (8.67, 12.93)
Tigray Region	2	8.53 (2.34, 19.39)
Over all pooled Result	11	33.93 (17.71, 50.14)

The finding of this study showed that 54.85% of pregnant women had identified the place for delivery (Table 3). The *I*^*2*^test result showed high heterogeneity (I^2=^99.9%, *p* < 0.001)and Eggers test (*p*-value < 0.001). Among the pregnant women included in this meta-analysis, only 64.06% planned to deliver at a health facility.

**Table 3:** Percent of pregnant women who identified place of deliveryas birth preparedness and complication readiness plan in Ethiopia

Study area	Number of studies	Estimation (95% CI)
SNNP Region	4	49.45 (0.61, 98.29)
Dire Dawa city	1	98.00 (96.64, 99.36)
Harari Region	1	57.20 (52.48, 61.92)
Oromia Region	2	61.27 (30.60, 91.94)
Amhara Region	1	44.60 (41.20, 48.00)
Tigray Region	2	40.86 (36.25, 45.47)
Over all pooled Result	11	54.85 (32.06, 77.64)

## Discussion

The aim of this systemic review and meta-analysis was to assess utilization of birth preparedness and complication readiness plan among women’s in Ethiopia. Thrteen studies were included in this meta-analysis.

Finding of this meta-analysis showed that only one-thirds of pregnant women in Ethiopia were prepared for birth and its complication. This finding is relatively similar to the study conducted in Uganda (35%) [39]. But it was Higher than a study carried out in Gambia (14%) [40]. In contrast, it is lower than studies carried out in West Bengal, India (57%) [41], Dehli, India (41%) [4] and Osun State, Nigeria (61%) [42].This difference might be due to socio-cultural issues like use of traditional birth attendant, women’s educational, economical status and the difference in implementation of prenatal health programs like quality of antenatal care services.

Similarly finding of this review reveals preparation of blood donor, awareness on danger signs and arrangement of transportation for emergency situation were among the poorly utilized elements of birth preparedness and complication readiness plan in Ethiopia.

Failure to recognize signs of complications is one the barriers for the delay in deciding to seek care [6, 43]. In this study 26.33% of the pregnant women were aware of danger signsof pregnancy. This result was consistent with a study conducted in Mpwapwa district, Tanzania [44] but it was less than study conducted in District of Ghana [45],West Bengal, India [41], and Abia State, southern Nigeria [46, 47]. In order to improve the awareness of women on danger signs of pregnancy the local and federal government should work intensively on empowering women’s education and economic level.

In addition the distance from woman’s home to a health facility and lack of transport during emergency are among the barriers for the delay in reaching a health facility. The result of this study showed only one third (33.93%) of pregnant women had arranged transportation for birth and emergency case; this is relatively consistent to study conducted in Burkina Faso (41.6%) [48]. But thisfinding was lower compared to studies carried out in Nepal (64.8%) [49], Kenya (81.9%) [50], Rural district of Ghana (53.3%) [45] and West Bengal, India (44.5%) [41]. This difference could be due to cultural difference like use home delivery, presence of traditional birth attendant as choice for delivery service and lack of awareness can hinder in reaching timely to health facilities. Unavailability of roads in some of the rural setups may be another reason.

Finding of this study also showed that only 54.85% of pregnant women identified the place of delivery. This result was lower than studies conducted in Punjab, India (90%) [51] and Osun State, Nigeria (87.5%) [42]. According to 2016 Ethiopian DHS institutional delivery was very low (26%) [9].This could be one of the major reasons for the low institutional delivery. In Ethiopia, lack of awareness on the importance of skilled health facility deliveries, cultural beliefs and transport challenges are among causes for high number of deaths during childbirth stated by officials [52]. Therefore health care providers working at community and antenatal care facility should improve awareness through strengthening information, education and counseling at institutional and community level so as to plan women’s their place of delivery.

Similarly in this study very few pregnant women (8.18%) also prepared potential blood donor for emergency case that may occurre during pregnancy and childbirth. This result was more or less consistent with the study conducted in Osun State, Nigeria(11.3%) [42] but lower than study carried out in Nairobi, Kenya (28.7%) [43] and Acra, Ghana (31.6%) [53]. Those womens which did not prepare potential blood donor are at higher risk of sever morbidity and mortality since bleeding is life threatening situation which needs prompt intervention [54, 55].Therefore womens and support persons should be aware of the need of preparing potential blood donor to prepare for birth and adressing complications.

Overall findings of this study reveals wide gap in ultilization of birth prepardness and complication readiness by women’s, this implies the high maternal and neonatal mortality in ethiopia might be related with this poor BPCR plan since poor awareness on danger signs, lack of preparing transport, lack of funds and lack of preparation of blood donor in the event of an emergency predominantly hinders access to emergency obstetric and newborn care (EmONC) in case of complications. Inaddition asystemic review and meta-analysis conducted in developing countries on Birth Preparedness and Complication Readiness (BPCR) interventions in reducing maternal and neonatal mortality showed high correlation between BPCR and maternal & neonatal mortality [56–58].

Therefore clinicians and policy makers should mobilize resources for improving women’s BPCR through ongoing training of women’s, developing guidelines for provision of appropriate BPCR and regular field supervision.

This meta-analysis didn’t address the factors affecting for this low utilization of BPCR, therefore researchers should conduct further studies in this area.

Strenght of this review: Since there was no similar study previously, this review and metaanalysis showed the national pooled image of birth prepardness and complication readiness plan of women’s in ethiopia. Strictly following PRISMA guide line and Joanna Briggs Institute Meta-Analysis of Statistics Assessment and Review Instrument (JBI-MAStARI) during critical appraisal was additional strenght of this systemic review and meta analysis.

Presence of high statistical heterogenity in most of the subanalysis were considered as weekness of this review. Inaddition our search was limited to articles published in English language.

## Conclusion

The level of birth preparedness and complication readiness among pregnant women in Ethiopia was found very low as every pregnant woman should be expected to prepare for birth and complication. Specifically, from the key elements of BPCR plan very low percent of pregnant women prepared potential blood donor for the emergency situation during pregnancy and childbirth. Therefore, the Ethiopia ministry of health, Regional health bureaus, health facilities and other stakeholders should work to improve women’s birth preparedness and complication readiness plan. We recommended further systematic review and metaanalysis on factors that affect birth preparedness and complication readiness plan of pregnant women in Ethiopia.

## Abbreviations

BPCR: Birth Preparedness and Complication Readiness
CI: Confidence Interval
JBI-MAStARI: Joanna Briggs Institute Meta-Analysis of Statistics Assessment and Review Instrument
MDG: Millennium Development Goals
PRISMA: Preferred Reporting Items for Systematic Reviews and Meta-Analyses
SE: Standard Error
SNNPR: South Nation and Nationalities People Region
WHO: World Health Organization
